# The problem of colorectal anastomosis safety

**DOI:** 10.1097/MD.0000000000018560

**Published:** 2020-01-10

**Authors:** Marius Kryzauskas, Eligijus Poskus, Audrius Dulskas, Augustinas Bausys, Matas Jakubauskas, Ugne Imbrasaite, Gabija Makunaite, Justas Kuliavas, Rimantas Bausys, Eugenijus Stratilatovas, Kestutis Strupas, Tomas Poskus

**Affiliations:** Faculty of Medicine, Vilnius University, Vilnius, Lithuania.

**Keywords:** air-leak test, anastomosis testing, anastomotic leakage, colorectal anastomosis, indocyanine green test, methylene blue test

## Abstract

**Introduction:**

Anastomotic leakage (AL) remains one of the most threatening complications in colorectal surgery with the incidence of up to 20%. The aim of the study is to evaluate the safety and feasibility of novel – trimodal intraoperative colorectal anastomosis testing technique.

**Methods and analysis:**

This multi-center prospective cohort pilot study will include patients undergoing colorectal anastomosis formation below 15 cm from the anal verge. Trimodal anastomosis testing will include testing for blood supply by ICG fluorescence trans-abdominally and trans-anally, testing of mechanical integrity of anastomosis by air-leak and methylene blue leak tests and testing for tension. The primary outcome of the study will be AL rate at day 60. The secondary outcomes will include: the frequency of changed location of bowel resection; ileostomy rate; the rate of intraoperative AL; time, taken to perform trimodal anastomosis testing; postoperative morbidity and mortality; quality of life.

**Discussion:**

Trimodal testing of colorectal anastomosis may be a novel and comprehensive way to investigate colorectal anastomosis and to reveal insufficient blood supply and integrity defects intraoperatively. Thus, prevention of these two most common causes of AL may lead to decreased rate of leakage.

**Study registration:**

Clinicaltrials.gov (https://clinicaltrials.gov/): NCT03958500, May, 2019.

## 1 Introduction

Anastomotic leakage (AL) remains one of the most threatening complications in colorectal surgery with the incidence of up to 20%. AL may be a life-threating complication, although, even if it is managed it results in poor oncologic outcomes, prolonged hospital stay and increased health care costs.^[1–3]^

The etiology of AL is still not fully clear, although, some risk factors have been suggested, including patient and disease related factors as well as surgical technique failure.^[4,5]^ Insufficient blood supply at the proximal or distal ends of anastomosis, tension on anastomosis and insufficient integrity of anastomosis are the main causes of technical failure and they may be modified intraoperatively if detected.^[5,6]^ Various tests to investigate mechanical integrity of anastomosis have been proposed.^[7–10]^ The most common test for colorectal anastomosis is an air-leak test. Some studies suggest saline or methylene blue leak tests alone or in combination with air-leak test as well.^[7]^ Although, these liquid based tests are much more common in gastrointestinal anastomoses and there is a lack of data for colorectal surgery.^[7,11–13]^ Intraoperative colonoscopy is another available method.^[8,14]^ As shown previously all of these tests for mechanical integrity reduce the rate of postoperative AL.^[7–10]^ However, it remains unclear whether some of them may be more accurate than others and which tests should be used. Moreover, negative results of integrity testing do not guarantee uneventful postoperative course.^[15]^ Insufficient blood supply is another well-known factor which is responsible for a postoperative leak of intraoperatively non-leaking anastomosis. Historically, the bowel viability and blood supply were evaluated by the surgeon through visual inspection. The color of the bowel wall, peristalsis of the bowel, pulsation of the marginal artery, or bleeding of the resected bowel margin is considered as clinical indicators of good vascularization.^[16]^ However, this is very subjective, and it does not always properly evaluate the micro-perfusion of the bowel wall. Intraoperative indocyanine green fluorescence angiography (ICG-FA) was proposed as more objective alternative which also accurately evaluates the micro-perfusion. Recently published study demonstrates very promising results for this technique, since AL rate was reduced twofold (3.5% vs 7.4%, respectively, *P* = .002) when ICG was used.^[17]^ Moreover, the usage of ICG in the study lead to change of the bowel resection place in 10.8% of patients.^[17]^ Similarly, De Nardi et al showed insufficient blood supply at the bowel requiring to extend the resection margin in 11% of patients, despite that this randomized controlled trial failed to show significantly reduced AL rate by using ICG.^[18]^ These results are encouraging, but as with isolated mechanical integrity testing, the isolated blood supply testing does not prevent all postoperative AL.

Therefore, we hypothesize that by using trimodal testing for mechanical integrity, blood supply and tension of anastomosis we can comprehensively evaluate anastomosis intraoperatively and to reduce the level of AL to minimum.

## 2 Methods

### 2.1 Study setting

This multi-center prospective cohort pilot study will be conducted at the 2 major colorectal surgery centers in Lithuania: Vilnius University hospital Santaros Klinikos and National Cancer Institute. The volume of these centers together is more than 600 colorectal resections annually.

### 2.2 Eligibility criteria

The study will include patients undergoing elective open or laparoscopic surgery for benign or malignant diseases of left-sided colon or rectum when the colorectal anastomosis will be below 15 cm from the anal verge by the rigid proctoscope. Patients over 18 years willing to participate and signing the informed consent will be included. Exclusion criteria will include the pregnancy and allergy to indocyanine green dye.

### 2.3 Sample size

Thirty patients will be included in the study since Kieser and Wassmer calculated that a pilot trial sample size between 20 and 40 would minimize the overall sample size for a main study sample size of 80 to 250 participants corresponding to standardized effect sizes of 0.4 and 0.7 (for 90% power based on a standard sample size calculation).^[19]^

### 2.4 Preoperative care

Preoperative patients’ preparation to surgery protocol will be standardized. Oral antibiotics (erythromycin 400 mg and metronidazole 500 mg) will be given 3 times on the day before surgery as well as oral mechanical bowel preparation. Low molecular weight heparin (nadroparin) will be administered 12 hours prior to surgery, according to patient's body weight. Standard preoperative antibacterial therapy (cefuroxime 1500 mg and metronidazole 500 mg) will be infused 30 minutes prior to incision.

### 2.5 Surgery and intraoperative testing of anastomosis

**Figure 1:**
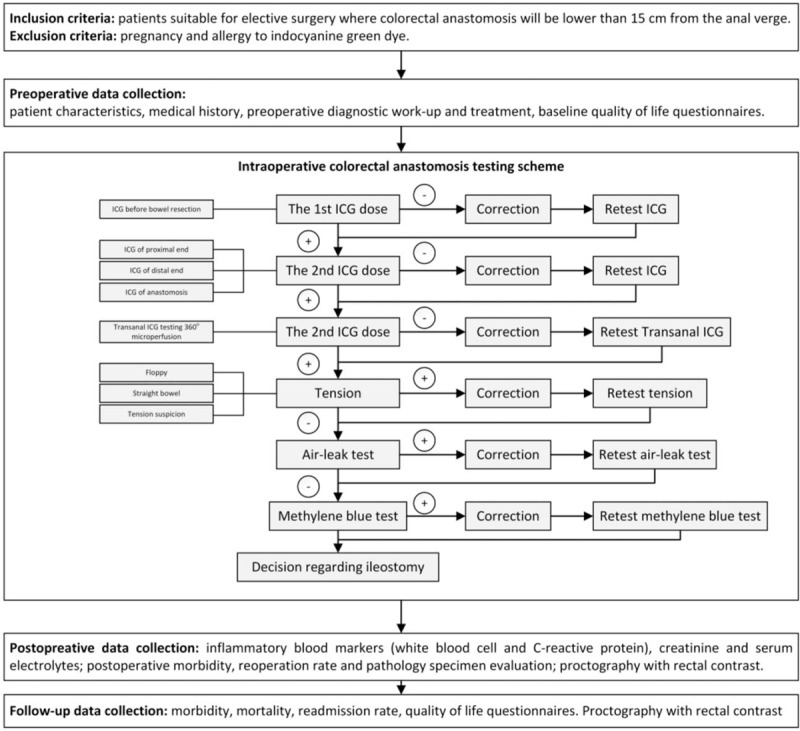
Detailed flowchart of the study.

Laparoscopic or open colorectal resection will be performed according to standard techniques of the study institutions. Trimodal anastomosis testing for blood supply, tension and mechanical integrity will be performed as shown in Figure 1.

ICG-FA tests will evaluate the micro- and macro-perfusion of the anastomosis at several timepoints.

Two doses of ICG dye will be prepared by diluting 25 mg of VERDYE ICG dye (Diagnostic Green, Aschheim, Germay) in 10 ml of sterile water. The first ICG (12.5 mg/5 ml) dose will be administered intravenously after division of the mesentery just before bowel resection to evaluate blood supply at the planned point of resection. The illumination of clearly visible arterial branches and subsequent illumination of bowel wall tissues will be considered as good perfusion and the resection of the bowel will be performed. If blood supply at planned point will be considered as insufficient the resection margin will be changed to the area of enough perfusion. After second ICG (12.5 mg/5 ml) injection just before anastomosis creation second fluorescence test will be performed just before and immediately after the creation of anastomosis to evaluate the proximal and distal parts of anastomosis.

If any segments of anastomosis will show a poor blood supply, it will be recreated or reinforced. Sometimes anastomosis will be deep in the pelvis and evaluation of the perfusion of anastomosis trans-abdominally is not possible, then n/a will be marked. After the satisfactory results of trans-peritoneal evaluation of perfusion by ICG, the additional trans-anal ICG testing will be performed by the technique described previously.^[20]^ Briefly, RECTOVISION proctoscope (Karl Storz, Tuttligen, Germany) for camera will be used, to ensure adequate trans-anal view air will be insufflated manually. The perfusion of the anastomosis from the mucosal side by fluorescence will be checked circumferentially. If any parts of anastomosis appear under perfused, corrections will be made.

Next, the tension on the anastomosis will be tested visually. We will aim at creating floppy anastomosis, whereby the bowel freely falls into the pelvis. The situation, where the bowel goes straight to the anastomosis but there is no obvious tension will be marked as straight anastomosis. If anastomosis appears to be under tension, corrections will be made.

Afterwards, mechanical integrity of the anastomosis will be tested by standard air-leak test through proctoscope. This will be performed simultaneously with the trans-anal perfusion testing under direct camera vision. The anastomosis should be under irrigation of saline solution in the pelvic cavity. The proximal colon is occluded by placing a soft bowel clamp across the bowel (without mesentery) comfortable distance above colorectal anastomosis. If air-leak test is positive, the leaking part of anastomosis will be reinforced. If leaking part is not identifiable temporary ileostomy will be created. Following air-leak test additional methylene blue leak test will be performed through a 16 French Foley catheter inserted in the anus. The catheter balloon will be inflated to 20 ml, avoiding the stretch of the anastomosis and gently withdrawn to the internal anal sphincter to avoid the spilling of staining solution. The volume of injected staining solution will depend on the height of colorectal anastomosis. This step is performed under direct laparoscopic (or open) vision to avoid stretching of anastomosis. In cases when low colorectal anastomosis will be impossible to visualize trans-abdominally, white gauze will be introduced and positioned around anastomosis before dye injection. If methylene blue leak test will be positive the defect in anastomosis will be repaired by reinforcing sutures; the decision to perform diverting ileostomy will be left to the surgeon.

### 2.6 Postoperative care

Postoperative care of the patients will be as per standard institutional protocol. The patients will be treated under the enhanced recovery after surgery (ERAS) protocol (early nutrition, early ambulation, early removal of catheter, prevention of nausea and vomiting, non-steroidal anti-inflammatory drug analgesia, no nasogastric tubes). The white blood cell count and C-reactive protein tests will be performed on the postoperative days 2, 4 and 6.^[21]^ Creatinine level and serum electrolyte tests will be performed on postoperative days 2 and 6. Proctography with water-soluble rectal contrast enema will be performed to check the integrity of colorectal anastomosis on postoperative day 7(±1). Digital rectal examination of very low anastomoses, endoscopy or computerized tomography with rectal contrast may be alternatives to evaluate the integrity of anastomosis when proctography will not be feasible.

### 2.7 Outcomes

The primary outcome of the study will be the AL at 60 days postoperatively. AL diagnosis will be made if clinical or radiological signs will be present. Proctography with water-soluble rectal contrast enema will be performed to check the integrity of anastomosis on postoperative day 7(±1) and 60(±7). In cases where proctography will not be feasible digital rectal examination, endoscopy or computerized tomography with rectal and/or oral contrast will be allowed as alternative methods. Fluid collection or abscess near the colorectal anastomosis will also be considered as AL.^[22]^

The secondary outcomes will include: the frequency of changed location of bowel resection after ICG testing; ileostomy rate; the rate of intraoperative AL; time, taken to perform trimodal anastomosis testing; postoperative morbidity and mortality; quality of life.

Quality of life will be assessed using low anterior resection syndrome (LARS) score and European Organization for Research and Treatment of Cancer Quality of Life Questionnaire Core-30 (EORTC QLQ-C30 (Version 3) before the operation and at day 60(±7).

## 3 Data collection and management

All the data will be recorded in a case report form. Data will be collected at preoperatively, intraoperatively, postoperatively during the intrahospital period and after discharge patients will be followed up until day 60. Data collected at various timepoints is shown in Table 1. Intraoperative anastomosis testing checklist used to evaluate the different aspects of the testing is shown in Table 2.

**Table 1:**
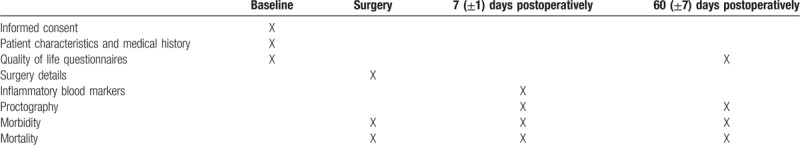
Data collection plan.

**Table 2:**
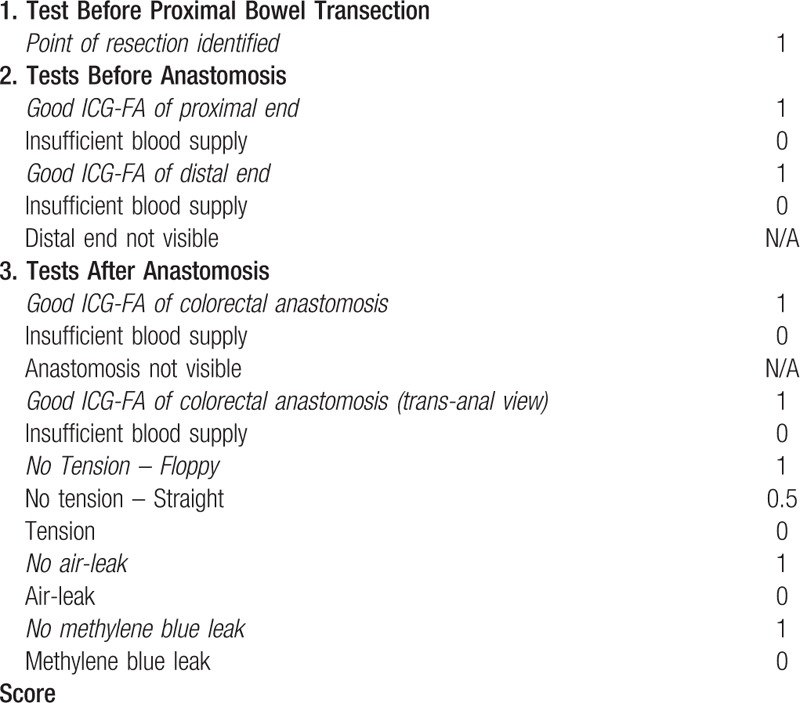
Checklist of intraoperative colorectal anastomosis testing.

### 3.1 Trial registration and ethical considerations

The study was approved by Vilnius Regional Bioethics Committee (Approval number 2019/3-116-608) and registered on Clinicaltrials.gov database (NCT03958500) on May 2019. Written informed consent will be obtained from the patients before participation in the study. The trial will be performed guided by World Medical Association's Declaration of Helsinki, Guideline for Good Clinical Practice, and regulatory laws in Lithuania.

## 4 Discussion

This pilot study will investigate the safety and feasibility of the comprehensive trimodal intraoperative testing of the blood supply, tension, and mechanical integrity of colorectal anastomosis.

The intraoperative blood supply, tension and mechanical integrity testing reduces the rate of postoperative AL when applied separately.^[23]^ Although, even if anastomosis is tested intraoperatively the rate of AL remains high.^[23,24]^ These 3 tests investigate different potential technical pitfals, therefore, there is a rationale to combine and perform all these tests together. Currently, there is a lack of studies investigating the potency of the multi-modal testing, therefore this study was designed. We hypothesize that trimodal testing will reduce the rate of postoperative AL to the minimal level by avoiding all the leakages due to technical failure and may identify the patients who can safely avoid preventive ileostomy.

Methylene blue leak test will be used for the mechanical integrity testing besides standard air-leak test. This test is cheap, safe and simple method to assess the integrity of anastomosis allowing the surgeon to identify leaking anastomosis at the time of surgery. There are no studies presenting methylene blue test as an additional technique for mechanical integrity testing in colorectal surgery to date and ours will be the first. Only few studies on methylene blue testing alone are published too. Smith et al reported the methylene blue leakage rate of 7% and 3.3% postoperative AL rate.^[7]^ It is important to mention that all postoperative AL happened to patients with negative methylene blue test intraoperatively. The authors concluded that methylene blue is feasible and easier to pinpoint the leak compared to air-leak test. Chen et al also presented similar results with a 14.5% of intraoperative leakage in patients undergoing rectal surgery.^[12]^ It seems, that methylene blue may have an advantage over other leak tests by easier identification of leakage site.^[6,8]^ In addition, the methylene blue testing is very safe, since no untoward effects have been described.^[7,11,25]^

We do not expect this test to be perfect in preventing leaks completely, it might be associated with reduction of the risk.^[7,12,26]^ The patients might have developed leaks after methylene blue test due to patient factors, insufficient blood supply, anastomotic tension and others.^[7,12,27]^ ITCORA study (ClinicalTrials.gov registry identifier NCT03316677) is the only one which compares air-leak test and methylene blue test.^[28]^ Nevertheless, this trial is not yet recruiting patients. By testing systematically the mechanical integrity, blood supply and tension of anastomosis we could account for these factors in the development of the leak.

Recently, ICG-FA was shown to reduce the risk of postoperative AL. Nevertheless, the majority of the studies were cohort studies with insufficient level of evidence.^[29–32]^ One of the first observational prospective study was PILLAR II trial by Jafari et al for colorectal anastomosis.^[33]^ The surgical plan was changed in 8% of the patients. The postoperative AL rate was 1.4%, but there were none in the group of patients who had a change in surgical plan based on ICG-FA. After encouraging results, PILLAR III (ClinicalTrials.gov registry identifier NCT02205307) randomized controlled trial was initiated to compare AL rate after standard and ICG-FA based colorectal resection.^[34]^ Unfortunately, PILLAR III study was terminated due to slow recruitment. There is only one published randomized controlled trial presented by De Nardi et al (ClinicalTrials.gov registry identifier NCT02662946).^[18]^ The bowel resection was extended due to insufficient perfusion of the colon stump for 11% of the patients. The reported AL rate was 5% and 9% in the ICG and control groups, respectively.

There are three ongoing randomized controlled trials. ICG-COLORAL study (ClinicalTrials.gov registry identifier NCT03602677) has planned to enroll 1062 participants where anastomosis perfusion is evaluated using ICG-FA as an addition to standard clinical practice compared to surgical practice alone.^[35]^ FLAG trial (ClinicalTrials.gov registry identifier NCT03390517) is similar to the one mentioned above.^[36]^ The investigators have planned to involve 300 participants and to compare colon and rectal tissue perfusion with ICG-FA and without this method. The primary outcome of this study is AL rate. Besides, the radiological anastomosis integrity will be checked on 7 to 8 postoperative day.

IntAct trial (ISRCTN.com registry identifier ISRCTN13334746) is also ongoing randomized controlled trial comparing surgery with ICG-FA against standard surgical practice.^[37]^ Moreover, there are 2 sub-studies which explore the role of the rectal microbiome in AL and the value of preoperative CT angiography and perfusion CT in predicting AL. Investigators consider it may help in understanding the mechanisms underlying AL.

None of these studies will systematically test the integrity and tension on the colorectal anastomosis, which, to our opinion, are important in the development of AL. We aim to develop an original, standardized, simple reproducible inspection method of colorectal anastomosis, which will systemically evaluate the colorectal anastomosis vascularity and mechanical integrity. We believe that combined evaluation should reduce the final AL risk.

Marius Kryzauskas orcid: 0000-0002-6373-9721.
